# Age Does Matter in Adolescents and Young Adults versus Older Adults with Advanced Melanoma; A National Cohort Study Comparing Tumor Characteristics, Treatment Pattern, Toxicity and Response

**DOI:** 10.3390/cancers12082072

**Published:** 2020-07-27

**Authors:** Monique K. van der Kooij, Marjolein J.A.L. Wetzels, Maureen J.B. Aarts, Franchette W.P.J. van den Berkmortel, Christian U. Blank, Marye J. Boers-Sonderen, Miranda P. Dierselhuis, Jan Willem B. de Groot, Geke A.P. Hospers, Djura Piersma, Rozemarijn S. van Rijn, Karijn P.M. Suijkerbuijk, Albert J. ten Tije, Astrid A.M. van der Veldt, Gerard Vreugdenhil, Michel W.J.M. Wouters, John B.A.G. Haanen, Alfonsus J.M. van den Eertwegh, Esther Bastiaannet, Ellen Kapiteijn

**Affiliations:** 1 Department of Medical Oncology, Leiden University Medical Center, Albinusdreef 2, PO box 9600, 2300 RC Leiden, The Netherlands; m.k.van_der_kooij@lumc.nl (M.K.v.d.K.); m.j.a.l.wetzels@umail.leidenuniv.nl (M.J.A.L.W.); 2 Department of Medical Oncology, Maastricht University Medical Center, P. Debyelaan 25, 6202 AZ Maastricht, The Netherlands; mjb.essers.aarts@mumc.nl; 3 Department of Medical Oncology, Zuyderland Medical Center, 6130 MB Sittard-Geleen, The Netherlands; f.vandenberkmortel@zuyderland.nl; 4 Department of Medical Oncology, Netherlands Cancer Institute- Antoni van Leeuwenhoek Hospital, Plesmanlaan 121, 1066 CX Amsterdam, The Netherlands; c.blank@nki.nl (C.U.B.); j.haanen@nki.nl (J.B.A.G.H.); 5 Department of Medical Oncology, Radboud University Medical Center, Geert Grooteplein Zuid 10, 6500 HB Nijmegen, The Netherlands; Marye.Boers-Sonderen@radboudumc.nl; 6 Department of Pediatric Oncology, Princess Máxima Center, Heidelberglaan 25, 3584 CS Utrecht, The Netherlands; M.P.Dierselhuis@prinsesmaximacentrum.nl; 7 Isala Oncology Center, Isala, 8000 GK Zwolle, The Netherlands; j.w.b.de.groot@isala.nl; 8 Department of Medical Oncology, University Medical Center Groningen, Hanzeplein 1, 9713 GZ Groningen, The Netherlands; g.a.p.hospers@umcg.nl; 9 Department of Medical Oncology, Medisch Spectrum Twente, Koningsplein 1, 7512 KZ Enschede, The Netherlands; D.Piersma@mst.nl; 10 Department of Medical Oncology, Medical Center Leeuwarden, Henri Dunantweg 2, 8934 AD Leeuwarden, The Netherlands; Rozemarijn.van.Rijn@ZNB.NL; 11 Department of Medical Oncology, University Medical Center Utrecht, Heidelberglaan 100, 3584 CX Utrecht, The Netherlands; K.Suijkerbuijk@umcutrecht.nl; 12 Department of Medical Oncology, Amphia Ziekenhuis, Langendijk 175, 4819 EV Breda, The Netherlands; AtenTije@amphia.nl; 13 Department of Medical Oncology, Erasmus MC Cancer Institute, Dr. Molewaterplein 40, 3000 CA Rotterdam, The Netherlands; a.vanderveldt@erasmusmc.nl; 14 Department of Medical Oncology, Maxima Medical Center, de Run 4600, 5500 MB Veldhoven, The Netherlands; g.vreugdenhil@mmc.nl; 15 Department of Surgical Oncology, Netherlands Cancer Institute- Antoni van Leeuwenhoek Hospital, Plesmanlaan 121, 1066 CX Amsterdam, The Netherlands; m.wouters@nki.nl; 16 Scientific Bureau, Dutch Institute for Clinical Auditing, Rijnsburgerweg 10, 2333 AA Leiden, The Netherlands; 17 Department of Medical Oncology, Cancer Center Amsterdam, Amsterdam UMC, Vrije Universiteit Amsterdam, de Boelelaan 1117, 1081 HZ Amsterdam, The Netherlands; vandeneertwegh@vumc.nl; 18 Department of Surgery, Leiden University Medical Centre, Albinusdreef 2, PO box 9600, 2300 RC Leiden, The Netherlands; E.Bastiaannet@lumc.nl

**Keywords:** advanced melanoma, adolescents, young adults, AYA, BRAF mutation, checkpoint inhibitors, targeted therapy, prospective nation-wide data, outcome research, clinical audit

## Abstract

Cutaneous melanoma is a common type of cancer in Adolescents and Young Adults (AYAs, 15–39 years of age). However, AYAs are underrepresented in clinical trials investigating new therapies and the outcomes from these therapies for AYAs are therefore unclear. Using prospectively collected nation-wide data from the Dutch Melanoma Treatment Registry (DMTR), we compared baseline characteristics, mutational profiles, treatment strategies, grade 3–4 adverse events (AEs), responses and outcomes in AYAs (*n* = 210) and older adults (*n* = 3775) who were diagnosed with advanced melanoma between July 2013 and July 2018. Compared to older adults, AYAs were more frequently female (51% versus 40%, *p* = 0.001), and had a better Eastern Cooperative Oncology Group performance status (ECOG 0 in 54% versus 45%, *p* = 0.004). BRAF and NRAS mutations were age dependent, with more BRAF V600 mutations in AYAs (68% versus 46%) and more NRAS mutations in older adults (13% versus 21%), *p* < 0.001. This finding translated in distinct first-line treatment patterns, where AYAs received more initial targeted therapy. Overall, grade 3–4 AE percentages following first-line systemic treatment were similar for AYAs and older adults; anti-PD-1 (7% versus 14%, *p* = 0.25), anti-CTLA-4 (16% versus 33%, *p* = 0.12), anti-PD-1 + anti-CTLA-4 (67% versus 56%, *p* = 0.34) and BRAF/MEK-inhibition (14% versus 23%, *p* = 0.06). Following anti-CTLA-4 treatment, no AYAs experienced a grade 3–4 colitis, while 17% of the older adults did (*p* = 0.046). There was no difference in response to treatment between AYAs and older adults. The longer overall survival observed in AYAs (hazard ratio (HR) 0.7; 95% CI 0.6–0.8) was explained by the increased cumulative incidence of non-melanoma related deaths in older adults (sub-distribution HR 2.8; 95% CI 1.5–4.9), calculated by competing risk analysis. The results of our national cohort study show that baseline characteristics and mutational profiles differ between AYAs and older adults with advanced melanoma, leading to different treatment choices made in daily practice. Once treatment is initiated, AYAs and older adults show similar tumor responses and melanoma-specific survival.

## 1. Introduction

Over the past decade, various systemic treatment options have become available for patients with advanced melanoma. These therapies include; antibodies targeting immune checkpoint T-lymphocyte-associated protein 4 (anti-CTLA-4), programmed cell death protein 1 (anti-PD-1) and targeted therapy against the BRAF kinases and MEK. In the Adolescent and Young Adult (AYA) population, defined as anyone between the ages of 15 and 39 years old, melanoma is less common when compared to adults older than 40 years of age. The 1-year incidence age-standardized risk for older adults in the Netherlands was 68.1 per 100,000 persons in 2018, when compared to 10.3 for AYAs. The same difference in 1-year incidence could be observed on a global scale; 8.7 per 100,000 persons in older adults versus 0.89 in AYAs [1]. Melanoma remains, however, one of the most frequently occurring cancers in AYAs, accounting for 4% of all cancers diagnosed in this age group [2].

Even though melanoma accounts for an important fraction of disease in AYAs, it is relatively uncommon in this age group when looking at the whole population. Therefore, only few patients are included in phase 3 studies. Current knowledge on prognostic factors and treatment strategies for (advanced) melanoma patients derives from these large phase 3 trials in patients with a median age varying between 53 and 62 [3,4,5,6,7,8] years of age. The relevance of the results from these trials for AYAs is therefore unclear. Over the past years, multiple differences in melanoma characteristics between the AYAs and (older) adults have been suggested. 

Daryanani et al. demonstrated that adolescents, defined as patients between 12 and 19 years of age, more often have locally advanced superficial spreading melanoma (SSM) as compared to adults. On the other hand, adults are more frequently diagnosed with nodular melanoma (NM) [9]. Indini et al. showed that melanoma was more common in male patients above 39 years of age, whereas females were more commonly afflicted with melanoma when under 39 years of age [10]. 

Mutations in genes encoding BRAF and NRAS proteins are the most common mutations found in melanoma, where approximately 50% of melanomas harbor a BRAF mutation and about 20% carry an NRAS mutation [11]. One of the most striking findings in smaller retrospective studies is that a higher incidence of BRAF mutations was observed in AYAs as compared to older adults [12], whereas NRAS mutations are more frequently detected in adults [13]. Differences in mutational profile and extent and manner of dissemination could influence the treatment choices made in daily clinical practice in AYAs versus older adults. Data on initiated treatments, efficacy and survival are currently lacking. 

Using prospectively collected real-life nation-wide data from the Dutch Melanoma Treatment Registry (DMTR), we had a unique opportunity to study this specific group in a large dataset. Our aim was to validate the previously described differences and similarities between melanomas that are detected in AYAs versus older patients of ≥40 years of age. First, we compared baseline characteristics of AYAs and older adults. Second, we compared treatment strategies, treatment-related AEs and progression-free survival (PFS). Additionally, we performed a competing risk analysis for non-melanoma-related death, next to overall survival (OS) and disease-specific survival (DSS) analyses. 

## 2. Results

### 2.1. Patient Selection and Baseline Characteristics

**Figure 1:**
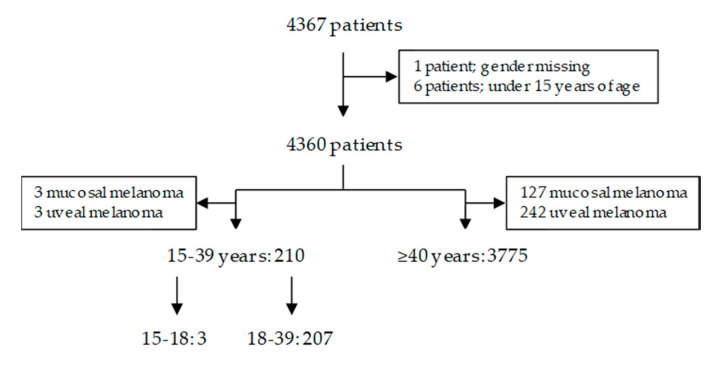
Patient selection for statistical analysis.

Between July 2013 and July 2018, 4367 patients were registered in the DMTR database. In total, 3985 advanced melanoma patients were eligible for analysis; 210 between 15 and 39 years of age (AYAs) (5%) versus 3775 older adult patients (95%). For details on patient selection, see Figure 1.

Differences in clinical and tumor characteristics between AYAs and older adults are shown in Table 1. Overall, AYAs were more frequently female, with a better ECOG performance status. Characteristics associated with tumor spread, including lactate dehydrogenase levels (LDH), Metastatic stage (M-stage) and the presence of brain metastases, were comparable between the two groups.

Characteristics of the primary melanoma are shown in the lower part of Table 1. AYAs had more SSM, while the primary was more frequently NM in older adults (*p* = 0.003). Furthermore, the primary tumor location was more often unknown in AYAs (23.3% versus 15.1%) or located in the head/neck region (18.1% versus 13.9%), while older adults had more primary melanomas on the trunk (38.0% versus 30.5%), *p* = 0.003. AYAs more often had thinner melanomas (Breslow thickness ≤2 mm) when compared to older adults, 42.9% versus 32.2%, *p* < 0.001.

### 2.2. Tumor Mutations

**Figure 2:**
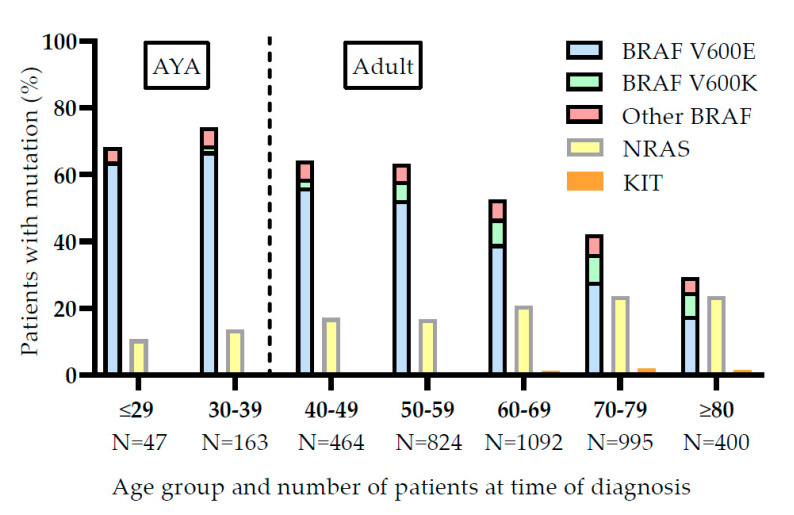
Incidence of BRAF, NRAS and KIT mutations in different age groups. Data on Adolescents and Young Adults (AYA) and older adults (Adult) is shown.

As shown in Table 1, AYAs more frequently harbor a BRAF V600 mutation (68.1% versus 45.6%), while older adults more frequently harbor an NRAS mutation (20.6% versus 12.9%) or have no BRAF V600 nor NRAS mutation (33.8% versus 19.0%), (*p* < 0.001). When further analyzing the percentage of patients having a BRAF V600 or NRAS mutation over different age groups, it was shown that the presence of a BRAF mutation is age dependent and shows a clear decrease of mutations over the 7 age groups in Figure 2. In the youngest AYA patient group between 15 and 29 years of age, 63.8% of patients harbored a BRAF V600 mutation, while of the patients of 80 years and older only 24.8% had this mutation.

Furthermore, the percentage of patients with an NRAS mutation increased with age. In the youngest patient group, 10.6% harbored an NRAS mutation, in patients of 80 years and over this was 23.5%. Overall, the incidence of KIT mutations was low (1.2%). There seemed to be a slight increase over age, from 0% to 1.5% in the oldest patient group. 

### 2.3. Initial Treatment 

**Figure 3:**
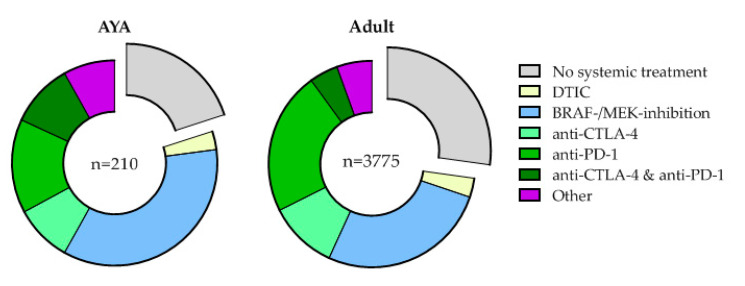
First-line melanoma treatment initiated in Adolescents and Young Adults (AYA) and older adults (Adult).

We analyzed the first-line treatment of all patients. Treatment patterns between AYAs and older adults differed significantly, *p* < 0.001. More AYAs were initially treated with BRAF/MEK-inhibition (35.2%) versus older adults (26.6%). Although the percentage of patients treated with immune checkpoint inhibitors did not differ between AYAs (33.8%) and older adults (37.6%), AYAs were given combination therapy with anti-PD-1 + anti-CTLA-4 more frequently (10.0% versus 4.5%), whereas monotherapy with anti-PD-1 was preferred in older adults (22.4% versus 14.8%), see Figure 3.

The initiation of BRAF/MEK-inhibition in AYAs remained constant over time since the start of our registry. Once anti-PD-1 was introduced, it largely replaced anti-CTLA-4 as first-line immune checkpoint inhibition in both AYAs and older adults. However, anti-PD-1 became more popular in older adults, while anti-CTLA-4 + anti-PD-1 was prescribed more to AYAs, see Figure S1. 

In AYAs, 42 patients (20.0%) did not receive any systemic treatment first-line versus 1027 older adults (27.2%). Twenty-nine AYAs underwent metastasectomy as first-line treatment versus 385 of the adults (69.0% versus 37.5%, *p* < 0.001). This difference was no longer significant after stratification for ECOG performance status, LDH level or M-stage. There was no difference in the percentage of patients receiving radiotherapy (33.3% versus 38.2%, *p* = 0.53). This treatment was given in the palliative setting in 64.3% of the AYAs and 50.3% in older adults. 

### 2.4. Treatment Toxicity

**Figure 4:**
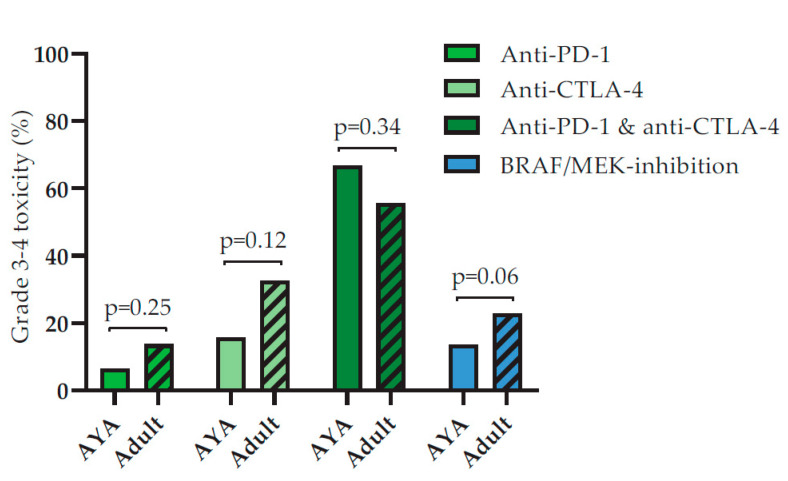
Toxicity rates following initial treatment for advanced melanoma of both Adolescents and Young Adults (AYAs) and older adults (Adult).

There was no difference in the occurrence of grade 3–4 AEs in AYAs or older adults for anti-PD-1 (6.5% versus 13.7%, *p* = 0.25), anti-CTLA-4 (15.8% versus 32.6%, *p* = 0.12) or anti-PD-1 with anti-CTLA-4 (66.7% versus 55.6%, *p* =0.34), nor following treatment with BRAF/MEK inhibitors (13.5% versus 22.8%, *p* = 0.06), see Figure 4. Data on types of grade 3–4 AEs is provided in Table S1.

Although colitis is one of the most frequently reported AEs following anti-CTLA-4 treatment in large phase 3 trials, AYAs did not seem to be affected. Following initial treatment with anti-CTLA-4 none of the 19 AYAs developed a grade 3–4 colitis, while 71 (17.4%) of the older adults did, *p* = 0.046. When we expanded the analysis, including all anti-CTLA-4 treated patients, regardless of the treatment line, we found that only 1 of the 48 (2.1%) AYAs experienced a grade 3–4 colitis. When we analyzed all older adults who received anti-CTLA-4 in any line of treatment, 134 of the 893 patients developed a 3–4 grade colitis (15.0%). Therefore, the difference in grade 3–4 colitis between AYAs and older adults remained significant, regardless of the timing of treatment, *p* = 0.013. 

Furthermore, AYAs more often developed a grade 3–4 hepatitis following anti-PD-1 and anti-CTLA-4 combination treatment when compared to older adults (9 out of 21 versus 36 out of 169, *p* = 0.03). The relatively high incidence of hepatitis in AYAs was only seen in the first line of treatment. When we analyzed all patients that were ever treated with combination checkpoint inhibition 26.4% of AYAs and 18.0% of older adults developed a hepatitis (*p* = 0.14).

### 2.5. Response to Systemic Treatment

We compared the best overall response (BOR) and overall response rates (ORR) between AYAs and older adults following either initial anti-PD-1, anti-CTLA-4, anti-PD-1 + anti-CTLA-4 or BRAF/MEK-inhibition, see Table 2. Although none of the treatment groups showed a difference in ORR, there was a difference in BOR between AYAs and older adults treated with initial anti-PD-1. AYAs more often had a complete response (CR) (38.7% versus 16.7%), while older adults had more partial response (PR) (35.6% versus 16.1%).

### 2.6. Survival

**Figure 5:**
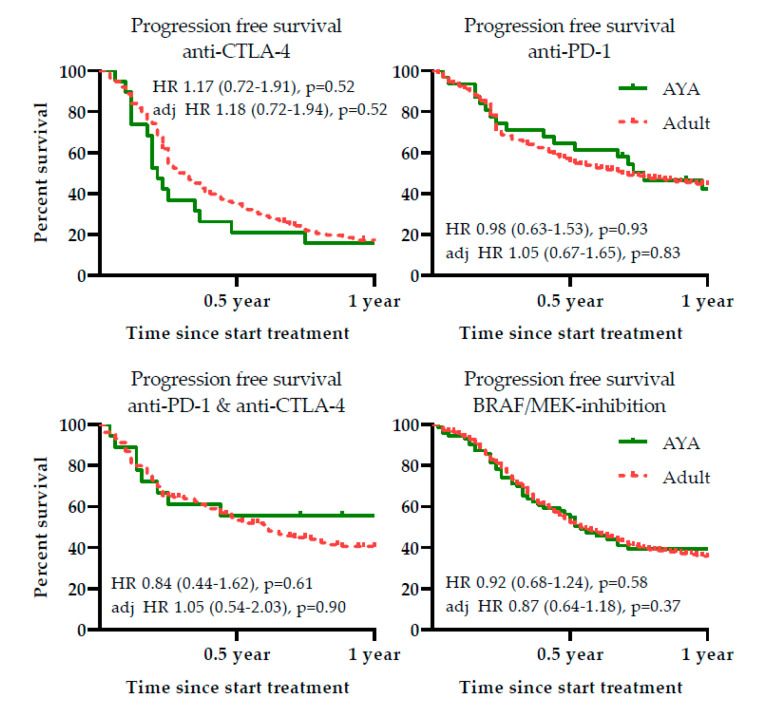
Progression free survival following first-line systemic therapy in Adolescents and Young Adults (AYAs) and older adult (Adult) patients. Hazard ratio (HR) for progression is provided, along with the adjusted HR (adjusted for: lactate dehydrogenase level, Eastern Cooperative Oncology Group performance status, distant metastasis in ≥3 organ sites, the presence of brain metastases and the presence of a BRAF V600 mutation).

There was no difference in PFS following systemic therapy in AYAs and older adults, see Figure 5. The 1-year PFS for anti-PD-1 was 42.2% (95% CI 23.8–60.6) in AYAs and 44.1% (95% CI 40.6–47.6) in older adults, *p =* 0.93. Following anti-CTLA-4 treatment, 1-year PFS of AYAs was 15.8% (95% CI 0–32.3) and 16.7% (95% CI 12.9–20.4) for older adults, *p* = 0.51. The combination treatment of anti-PD-1 and anti-CTLA-4 yielded a 1-year PFS in AYAs of 50.0% (95% CI 28.0–72.0) and 40.2% (95% CI 32.0–48.4) in older adults, *p =* 0.60. 

There were also no differences in PFS following targeted therapy: 1-year PFS in AYAs was 38.8% (95% CI 27.2–50.4), and 35.0% (95% CI 31.9–38.1) in older adults, *p* = 0.58. In Figure 5, both the crude HR for progression and adjusted HR are shown.

There was an OS advantage of AYAs over older adults, with a 1-year survival of 64.7% (95% CI 58.0–71.4) versus 55.0% (95% CI 53.4–56.6), *p* < 0.001. The HR for OS in AYAs was 0.69 (95% CI 0.57–0.84, *p* < 0.001) as compared to older adults (Table 3). The OS benefit of AYAs was higher in patients without a BRAF V600 mutation. However, even after adjusting for known prognostic factors (including BRAF V600 mutation) the influence of age group remained significant; adjHR 0.68 (95% CI 0.56–0.83, *p* < 0.001).

In AYAs 88.2% of all deaths was caused by melanoma, this was 75.1% in older adults, *p* = 0.002. DSS and a competing risk analysis were performed to further investigate the difference in OS between AYAs and older adults. The DSS was better in AYAs (HR 0.81; 95% CI 0.66–1.00 and adjHR 0.79; 95% CI 0.64–0.98). The competing risk analysis showed that the difference in OS between AYAs and adults could be explained by the occurrence of more non-melanoma-related deaths in the older patient group. When accounting for non-melanoma related death as a competing risk, there was no difference between AYAs and older adults; sub-distribution HR (sHR) 0.90 (95% CI 0.73–1.11), adjusted sHR 0.92 (95% CI 0.75–1.13). 

When addressing the non-melanoma-related deaths, both the non-melanoma specific survival (nMSS) and the sHR for melanoma specific death in AYAs as compared to older adults were lower. This indicated that AYAs had a significantly lower HR and sHR of dying of a non-melanoma-related cause; HR 0.32 (95% CI 0.18–0.57) and adjHR 0.33 (95% CI 0.18–0.58), sHR 0.36 (95% CI 0.20–0.63) and adjusted sHR 0.37 (95% CI 0.21–0.67). In Table 3 both the crude and adjusted HR for survival of AYAs and older adults is shown.

## 3. Discussion

In the largest prospective cohort study thus far, we observed that on a nation-wide scale 5% of all advanced melanoma patients were AYAs. Furthermore, we showed that BRAF and NRAS mutations are age dependent, leading to more AYAs being treated with targeted therapy. As current treatment strategies for this age group are adapted from clinical trials that mostly include older adults, it is important to investigate advanced melanoma in this young patient group [3,4].

By studying baseline characteristics of AYAs and older adults, we found more female patients in the AYA group than in the older adult group. This could be explained by both biological gender differences [14,15] and behavior differences between male and female patients. Donley et al. recently suggested that an early age at menarche and a late age at menopause are associated with an increased risk of melanoma in postmenopausal women [16]. An earlier study by Smith et al. and a recent study by Støer et al., however, did not find convincing evidence that reproductive factors are associated with an increased risk of melanoma [17,18]. As a result that the women in these studies had a median age of 53.5 (Smith et al.) and 48 years (Støer et al.), future research might focus on reproductive factors and exogenous estrogen use in younger women to try to explain why advanced melanoma is more abundant in women than in men within AYAs, when compared to older adults.

We determined a significant difference in the distribution of histological subtype of melanoma between the two age groups of interest. AYAs more often presented with SSM as compared to older adults, whereas older adults more frequently presented with NM. This is in accordance to what Verzì et al., Bartenstein et al. and Daryanani et al., previously described, although in cohorts with younger patients [9,19,20]. Moreover, we found that AYAs had significantly thinner tumors than older adults. Relatively more AYAs had a tumor with a Breslow depth below 2 mm, suggesting that these tumors were nevertheless more aggressive as they did develop into advanced disease. From earlier research we know that melanomas of patients under the age of 20 years were significantly thicker than the melanomas seen in their adult control group [21]. This difference could be explained by the relatively small number of patients under 20 years of age in our AYA group (*n* = 6).

Our analysis of mutational profiles revealed that the frequency of a BRAF V600 mutation declines with age, whereas the frequency of an NRAS mutation increases with age. Our findings support previous studies that found BRAF mutations to be more abundant in the AYAs than in older adults. A possible explanation is that melanomas without BRAF mutations require accumulation of high UV doses over time, further supported by the difference in anatomic site of primary tumor [13,22].

Moreover, AYAs were treated significantly more often with combination therapy (anti-CTLA-4 + anti-PD-1) than older adults. These differences might be explained by less fear of AEs in AYAs. However, we did not find differences in occurrence of grade 3–4 AEs between AYAs and older adults following immune checkpoint inhibition or BRAF/MEK-inhibition. 

Interestingly, there was a difference in toxicity pattern following anti-CTLA-4 monotherapy and in combination with anti-PD-1. Following combination treatment, more AYAs developed a hepatitis when compared to older adults (9 out of 21 versus 36 out of 169). None of the 19 AYAs developed a colitis following initial anti-CTLA-4 monotherapy, while 71 out of 408 older adults did. It has been suggested that the gut microbiota can influence the occurrence of treatment-related AEs and even treatment efficacy after checkpoint inhibitor therapy [23]. The Bacteroidetes phylum of the intestinal microbiota has been identified to be protective against anti-CTLA-4-induced colitis by stimulating the differentiation of regulatory T-cells, thereby limiting inflammation [24]. The intestinal microbiota changes throughout the human lifetime. It is unclear, however, whether AYAs have a higher proportion of Bacteroidetes than older adults, as recent studies have shown contradicting results [25,26]. Based on our findings, we encourage researchers of the intestinal microbiota to incorporate age dependent differences in their results. 

Increased age is associated with changes in host immunity that could impact the effectiveness of checkpoint inhibition. With advancing age, the immune system remodels and declines, predisposing older adults and elderly to a higher risk of infections, auto-immune diseases and malignancies as compared to younger adults [27]. The naïve T-cell compartment declines 2-5-fold between the ages of 30 and 70 years old, and the ability to establish immunological memory to newly introduced antigens is compromised [28,29]. Furthermore, it was shown that CD4+ T-cells of patients ≤ 50 show more signs of activation, when compared to patients ≥ 65 years of age [30]. These factors can contribute to a less effective T-cell immune response against melanoma cells after immunotherapy with checkpoint inhibition in older adults and elderly as compared to the younger population. However, we established that there was no difference in response, nor PFS between AYAs and older adults following immune checkpoint inhibition or targeted therapy. In addition, we showed that the favorable OS of AYAs as compared to older adults is due to an increased risk for adults to die from a non-melanoma-related cause.

Strengths of our dataset include the fact that all data are prospectively registered by trained data managers and is subsequently approved by the treating physician. Moreover, our analysis is based on nation-wide data. However, there are some limitations. During data collection treatment patterns for patients with advanced melanoma have changed. In the early stages of the DMTR dataset, tumor mutational status was not determined in all patients. Additionally, we might not have included all patients between 15 and 18 years of age, as some might not have been referred to the medical oncologist. Furthermore, we relied on data registered in the DMTR database, which was mostly of clinical origin. It would be interesting to combine the clinical results with data on immunological parameters in both the blood, tumor and stroma. Additionally, we did not have supplementary information on specific mutation signatures or whole genome sequencing data to compare AYAs with older adults. In accordance with our current data, both Krauthammer and Wilmott report a high frequency of BRAF mutations in younger patients, while adults more frequently harbor NRAS mutations. Furthermore, Krauthammer et al. showed that the presence of NF1 and RASA2 mutations was also age dependent. The presence of NF1 mutations was associated with a UV-derived mutation signature, higher mutational load and lower disease-specific survival [31]. The article by Willmott et al. published whole genome sequencing data from 25 Australian AYA and 121 adult patients [13]. They found high mutational signatures of ultraviolet radiation damage in all patients. It would be interesting to compare whole genome sequencing data from AYAs and older adults from the Netherlands, or another non-high ambient ultraviolet radiation country, with the data from Australia. This could shed more light on the reasons why AYAs develop advanced melanoma at a younger age, and which (distinct) signaling pathways are used.

## 4. Materials and Methods

### 4.1. Data Source

Advanced melanoma patients in the Netherlands, irrespective of the type of primary melanoma, are registered in the DMTR following referral to one of the 14 expert hospitals in the Netherlands. The nation-wide centralization of advanced melanoma patients and their registration in the DMTR was initiated in 2012 to assure safety and quality of melanoma care [32]. Up till May 2018, patients between 15 and 18 years of age were mostly treated at the previously mentioned 14 expert hospitals. Since then, all children <18 years of age are referred to the department of Pediatric Oncology at the Princess Máxima Center. Information on patient and tumor characteristics, treatment regimens, grade 3–4 AEs (according to the Common Terminology Criteria for Adverse Events, version 4.0) and clinical outcomes have since been entered into the DMTR. Data are collected from patient files by trained data managers and approved by the treating physicians. The study was conducted in accordance with the Declaration of Helsinki. In compliance with Dutch regulations, the DMTR was approved by a medical ethical committee (METC Leiden University Medical Center, 2013) and is not considered subject to the Medical Research Involving Human Subjects Act.

### 4.2. Patients

Between July 2013 and July 2018, 4367 patients with advanced melanoma were registered in the DMTR, follow-up data cut-off was set at March 1st, 2019. The patients with missing data on gender (*n* = 1) and patients under the age of 15 years old (*n* = 6) were excluded from the analysis. Furthermore, patients with mucosal and uveal melanoma were excluded from analysis (*n* = 375). After including all eligible patients, 3985 patients were analyzed according to their age at registration of advanced melanoma. Treatment strategies were categorized as systemic therapy and non-systemic treatment. Systemic therapy was further subdivided into chemotherapy (Dacarbazine/DTIC), immune checkpoint inhibition (anti-CTLA-4, anti-PD-1 or a combination of both), targeted therapy (BRAF-inhibition with or without MEK-inhibition) and ‘’other’’ systemic therapy. Treatment strategies could have been initiated either as standard care or in the context of participation in a clinical trial. Non-systemic treatment was further subdivided into metastasectomy (systemic therapy ‘’no’’, surgery ‘’yes’’ and metastatic lesion identified) and radiotherapy (palliative yes versus no). Differences in treatment pattern between AYAs and older adults were assessed using a Pearson’s chi-square test. In adherence with international guidelines and literature, AYAs were defined as all patients between 15 and 39 years of age [33].

### 4.3. Statistical Analysis

Patients were classified according to age group for all analyses; AYAs versus older adults. Patient baseline characteristics, type of primary tumor, localization of metastases (based on TNM 7th edition [34]), number of organ sites involved and frequencies of systemic therapy administration were determined using descriptive statistics. The difference between categorical variables for the different age groups was tested with a Pearson’s chi-square test. A median test for independent medians tests was used to compare the time from primary diagnosis until advanced disease.

A univariable Cox analysis using the variables “gender” (male versus female), “ECOG performance status” (ECOG 0, ECOG 1 or ECOG 2), “LDH level” (not elevated, elevated within 2× upper limit of normal or strongly elevated >2× upper limit of normal), “brain metastases” (yes versus no), “distant metastasis in ≥3 organ sites” (yes versus no), “histologic type of melanoma” (superficial spreading versus nodular and versus other), “location primary tumor” (unknown primary, head-and-neck region, trunk, extremities or acral) and “BRAF mutation” (whether a BRAFV600E or BRAFV600K mutation was present versus absent) was performed. Subsequently, a multivariable Cox regression model was estimated, including the following prognostic factors; LDH level, ECOG performance status, distant metastasis in ≥3 organ sites, the presence of brain metastases, BRAF mutation [35,36,37]. The histologic subtype of primary melanoma (specifically superficial spreading and nodular) was not added to the multivariable analysis as the prognostic value in the advanced setting seems limited. A recent study showed no difference in survival between these subtypes following immune checkpoint inhibition. Nodular melanoma had a worse prognosis following targeted therapy. However, LDH level, ECOG performance status and number of metastases were not added in their multivariate analysis [38].

To compare the safety of initial systemic treatment between AYAs and older adults, all included patients received at least one infusion/treatment with anti-CTLA-4, anti-PD-1, anti-CTLA-4 and anti-PD1 or BRAF/MEK inhibition. Differences in grade 3–4 toxicity were tested with a Pearson’s chi-square test.

Response evaluation in this uncontrolled real-world setting was based on clinical judgement of the medical team and was partially based on the RECIST 1.1 criteria. The BOR is the best evaluation that a patient received after initiation of treatment, until the start of a new melanoma therapy, or last visit at the treating physician; CR, PR, SD or PD. The ORR is defined by clinicians as having CR and PR. Differences in ORR between AYAs and older adult patients was tested with a Pearson’s chi-square test. Due to the limited number of AYAs in the different response groups, no test was performed to assess the possible statistical difference in BOR. 

OS, PFS and DSS were used to estimate survival probabilities [39]. As it is known that younger patients have a longer life expectancy, we used the cumulative incidence competing risk method (CIRC) to estimate melanoma-related mortality risk. To estimate survival, cumulative incidence curves with non-melanoma-related death as competing risk were used. To estimate sHR and corresponding 95% CI, Fine and Gray competing risk models were used with melanoma-related death as event and non-melanoma-related death as competing risk. Follow-up started at first visit after the diagnosis of advanced disease [40,41].

All statistical analyses were conducted using SPSS (IBM Corp. Released 2017. IBM SPSS Statistics for Windows, Version 25.0. IBM Corp, Armonk, NY, USA) and STATA (StataCorp. 2015. Stata Statistical Software: Release 14.1. College Station, StataCorp LP, Lakeway Drive College Station, TX, USA).

## 5. Conclusions

Concluding, we show, for the first time, on a nation-wide scale and with real-life data that the frequency of a BRAF V600 mutation declines with age, whereas the frequency of an NRAS mutation increases with age in patients with advanced melanoma. Furthermore, AYAs with advanced stage melanoma are more commonly afflicted with the histologic subtype of superficial spreading melanoma and have thinner tumors than older adults. Moreover, first-line treatment for AYAs is more often BRAF/MEK-inhibition or the combination of anti-CTLA-4 + anti-PD-1. These treatments lead to similar responses in both AYAs and older adults. Interestingly, toxicity patterns in AYAs were distinct with regard to anti-CTLA-4 monotherapy and combination treatment with anti-PD-1. Our study is an important step towards a better understanding of advanced melanoma in AYAs and might open doors for new studies with AYAs in order to improve daily clinical practice for advanced melanoma in the younger population.

## Figures and Tables

**Table 1:** Clinical and tumor characteristics of AYAs and older adult with advanced melanoma, and their primary melanomas. Eastern Cooperative Oncology Group (ECOG), lactate dehydrogenase (LDH), metastatic stage (M-stage).

Characteristic	AYA	Older adult	*p*-Value
Patients; *n*	210	3775	
Median age, year (range)	34 (15–39)	65 (40–97)	
**Gender; *n* (%)**			0.001
Male	102 (48.6)	2261 (59.9)	
Female	108 (51.4)	1514 (40.1)	
**ECOG PS; *n* (%)**			0.004
0	114 (54.3)	1694 (44.9)	
1	46 (21.9)	1090 (28.9)	
≥2	17 (8.1)	513 (13.6)	
Unknown	33 (15.7)	477 (12.6)	
**LDH; *n* (%)**			0.72
Normal	124 (59.0)	2171 (57.5)	
Elevated (<2xULN)	40 (19.0)	817 (21.6)	
High (≥2xULN)	30 (14.3)	472 (12.5)	
Unknown	16 (7.6)	315 (8.3)	
Metastasis in ≥3 organ sites; *n* (%)	68 (32.4)	1239 (32.8)	0.90
**M-stage; ***n*** (%)**			0.28
M1a	22 (10.5)	444 (11.8)	
M1b	15 (7.1)	403 (10.7)	
M1c	169 (80.5)	2829 (74.9)	
Unknown	4 (1.9)	99 (2.6)	
Brain metastasis; *n* (%)	60 (28.6)	1053 (27.9)	0.83
Symptomatic	42 (70.0)	715 (68.0)	0.74
**Mutational profile; *n* (%)**			<0.001
BRAF V600 mutation	143 (68.1)	1721 (45.6)	
BRAF V600E mutation	140 (66.7)	1466 (38.8)	
BRAF V600K mutation	3 (1.4)	255 (6.8)	
NRAS mutation	27 (12.9)	777 (20.6)	
No BRAF V600 or NRAS	40 (19.0)	1277 (33.8)	
**Type of primary melanoma; *n* (%)**			0.003
Superficial spreading	101 (48.1)	1535 (40.7)	
Nodular	26 (12.4)	832 (22.0)	
Other/unknown	83 (39.5)	1408 (37.3)	
**Location primary melanoma; *n* (%)**			0.003
Unknown primary	49 (23.3)	571 (15.1)	
Head and neck	38 (18.1)	525 (13.9)	
Trunk	64 (30.5)	1433 (38.0)	
Extremities	55 (26.2)	1142 (30.3)	
Acral	4 (1.9)	104 (2.8)	
**Breslow thickness; *n* (%)**			<0.001
≤2mm	90 (42.9)	1214 (32.2)	
2–4mm	43 (20.5)	943 (25.0)	
>4mm	20 (9.5)	754 (20.0)	
Unknown	57 (27.1)	864 (22.9)	

**Table 2:** Best overall response and objective response rate following systemic treatment in Adolescents and Young Adults (AYAs) and older adults. Best overall response (BOR) was classified as either; progressive disease (PD), stable disease (SD), partial response (PR) or complete response (CR). Objective response rate (ORR) was the combination of PR and CR.

**Anti-PD-1**		**AYAs (*n* = 31)**	**Older adults (*n =* 779)**	***p*-Value**
	PD	9 (29.0)	196 (25.2)	
	SD	5 (16.1)	176 (22.6)	
	PR	5 (16.1)	277 (35.6)	
	CR	12 (38.7)	130 (16.7)	
	ORR	17 (54.8)	407 (52.2)	0.78
**Anti-CTLA-4**		**AYAs (*n* = 17)**	**Older adults (*n* = 385)**	
	PD	10 (58.8)	166 (43.1)	
	SD	5 (29.4)	143 (37.1)	
	PR	1 (5.9)	45 (11.7)	
	CR	1 (5.9)	31 (8.1)	
	ORR	2 (11.8)	76 (19.7)	0.42
**Anti-PD-1 and anti-CTLA-4**		**AYAs (*n* = 18)**	**Older adults (*n* = 146)**	
	PD	8 (44.4)	35 (24.0)	
	SD	0	28 (19.2)	
	PR	7 (38.9)	69 (47.3)	
	CR	3 (16.7)	14 (9.6)	
	ORR	10 (55.6)	83 (56.8)	0.92
**BRAF/MEK inhibitor**		**AYAs (*n* = 68)**	**Older adults (*n* = 923)**	
	PD	15 (22.1)	148 (16.0)	
	SD	12 (17.6)	272 (29.5)	
	PR	36 (52.9)	452 (49.0)	
	CR	5 (7.4)	51 (5.5)	
	ORR	41 (60.3)	503 (54.5)	0.35

**Table 3:** Overall survival, disease specific survival and competing risk analyses of all advanced melanoma patients.

	Events (n)		Crude HR			Adjusted HR		
	AYA	Adult	HR	95% CI	*p*-Value	HR	95% CI	*p*-Value
OS	102	2292	0.69	0.57–0.84	<0.001	0.68	0.56–0.83	<0.001
DSS	90	1728	0.81	0.66–1.00	0.06	0.79	0.64–0.98	0.03
Competing Risk	90	1728	0.90 *	0.73–1.11	0.32	0.92 *	0.75–1.13	0.43
**Non-Melanoma**								
nMSS	12	574	0.32	0.18–0.57	<0.001	0.33	0.18–0.58	<0.001
Competing risk	12	574	0.36 *	0.20–0.63	<0.001	0.37 *	0.21–0.67	<0.001

Data on Cox proportional hazard model for overall survival (OS), disease specific survival (DSS), non-melanoma specific survival (nMSS) and Fine and Gray cause-specific cumulative incidence of death (competing risk) is shown. Number of deaths is shown (events) per age group; Adolescents and Young Adult (AYA) versus older adults (Adult). Crude hazard ratio (HR), and adjusted HR are shown. HR were adjusted for: lactate dehydrogenase level, Eastern Cooperative Oncology Group performance status, distant metastasis in ≥3 organ sites, brain metastases and the presence of a BRAF V600 mutation. * Sub-distribution HR, from the Fine and Gray model.

## Supplementary Materials

The following are available online at https://www.mdpi.com/2072-6694/12/8/2072/s1, Table S1. Grade 3–4 toxicity following initial systemic treatment. Subtypes of adverse events per treatment type are shown for Adolescents and Young Adults (AYA) and older adults (Adult). Figure S1: Types of initial systemic treatment initiated since the diagnosis of advanced melanoma. Cumulative number of targeted therapy and immune checkpoint inhibition initiated over time since July 2013 for Adolescents and Young Adults (AYA, solid line) and older adults (Adult, dotted line) is shown.

## References

[1] GLOBOCAN 2012 v1.0, Cancer Incidence and Mortality Worldwide: IARC CancerBase No. 11. http://globocan.iarc.fr.

[2] Siegel R.L., Miller K.D., Jemal A. (2019). Cancer statistics, 2019. CA. Cancer J. Clin..

[3] Hauschild A., Grob J.-J., Demidov L.V., Jouary T., Gutzmer R., Millward M., Rutkowski P., Blank C.U., Miller W.H., Kaempgen E. (2012). Dabrafenib in BRAF-mutated metastatic melanoma: A multicentre, open-label, phase 3 randomised controlled trial. Lancet.

[4] Chapman P.B., Hauschild A., Robert C., Haanen J.B., Ascierto P., Larkin J., Dummer R., Garbe C., Testori A., Maio M. (2011). Improved Survival with Vemurafenib in Melanoma with BRAF V600E Mutation. N. Engl. J. Med..

[5] Wolchok J.D., Chiarion-Sileni V., Gonzalez R., Rutkowski P., Grob J.-J., Cowey C.L., Lao C.D., Wagstaff J., Schadendorf D., Ferrucci P.F. (2017). Overall Survival with Combined Nivolumab and Ipilimumab in Advanced Melanoma. N. Engl. J. Med..

[6] Carlino M.S., Long G.V., Schadendorf D., Robert C., Ribas A., Richtig E., Nyakas M., Caglevic C., Tarhini A., Blank C. (2018). Outcomes by line of therapy and programmed death ligand 1 expression in patients with advanced melanoma treated with pembrolizumab or ipilimumab in KEYNOTE-006: A randomised clinical trial. Eur. J. Cancer.

[7] Hamid O., Puzanov I., Dummer R., Schachter J., Daud A., Schadendorf D., Blank C., Cranmer L.D., Robert C., Pavlick A.C. (2017). Final analysis of a randomised trial comparing pembrolizumab versus investigator-choice chemotherapy for ipilimumab-refractory advanced melanoma. Eur. J. Cancer.

[8] Larkin J., Minor D., D’Angelo S., Neyns B., Smylie M., Miller W.H., Gutzmer R., Linette G., Chmielowski B., Lao C.D. (2018). Overall Survival in Patients With Advanced Melanoma Who Received Nivolumab Versus Investigator’s Choice Chemotherapy in CheckMate 037: A Randomized, Controlled, Open-Label Phase III Trial. J. Clin. Oncol..

[9] Daryanani D., Plukker J.T., Nap R.E., Kuiper H., Hoekstra H.J. (2006). Adolescent melanoma: Risk factors and long term survival. Eur. J. Surg. Oncol..

[10] Indini A., Brecht I., Del Vecchio M., Sultan I., Signoroni S., Ferrari A. (2018). Cutaneous melanoma in adolescents and young adults. Pediatr. Blood Cancer.

[11] Seynnaeve B., Lee S., Borah S., Park Y., Pappo A., Kirkwood J.M., Bahrami A. (2017). Genetic and Epigenetic Alterations of TERT Are Associated with Inferior Outcome in Adolescent and Young Adult Patients with Melanoma. Sci. Rep..

[12] Menzies A.M., Haydu L.E., Visintin L., Carlino M.S., Howle J.R., Thompson J.F., Kefford R.F., Scolyer R.A., Long G.V. (2012). Distinguishing clinicopathologic features of patients with V600E and V600K BRAF-mutant metastatic melanoma. Clin Cancer Res..

[13] Wilmott J.S., Johansson P.A., Newell F., Waddell N., Ferguson P., Quek C., Patch A.-M., Nones K., Shang P., Pritchard A.L. (2019). Whole genome sequencing of melanomas in adolescent and young adults reveals distinct mutation landscapes and the potential role of germline variants in disease susceptibility. Int. J. Cancer.

[14] Hajdarevic S., Schmitt-Egenolf M., Brulin C., Sundbom E., Hörnsten Å. (2011). Malignant melanoma: Gender patterns in care seeking for suspect marks. J. Clin. Nurs..

[15] Giblin A.-V., Thomas J.M. (2007). Incidence, mortality and survival in cutaneous melanoma. J. Plast. Reconstr. Aesthetic Surg..

[16] Donley G.M., Liu W.T., Pfeiffer R.M., McDonald E.C., Peters K.O., Tucker M.A., Cahoon E.K. (2019). Reproductive factors, exogenous hormone use and incidence of melanoma among women in the United States. Br. J. Cancer.

[17] Smith M.A., Fine J.A., Barnhill R.L., Berwick M. (1998). Hormonal and reproductive influences and risk of melanoma in women. Int. J. Epidemiol..

[18] Støer N.C., Botteri E., Ghiasvand R., Busund M., Vangen S., Lund E., Veierød M.B., Weiderpass E. (2019). Reproductive factors and risk of melanoma: a population-based cohort study. Br. J. Dermatol..

[19] Verzì A.E., Bubley J.A., Haugh A.M., Zhang B., Wagner A., Kruse L., West D.P., Wayne J., Guitart J., Gerami P. (2017). A single-institution assessment of superficial spreading melanoma (SSM) in the pediatric population: Molecular and histopathologic features compared with adult SSM. J. Am. Acad. Dermatol..

[20] Bartenstein D.W., Kelleher C.M., Friedmann A.M., Duncan L.M., Tsao H., Sober A.J., Hawryluk E.B. (2018). Contrasting features of childhood and adolescent melanomas. Pediatr. Dermatol..

[21] Livestro D.P., Kaine E.M., Michaelson J.S., Mihm M.C., Haluska F.G., Muzikansky A., Sober A.J., Tanabe K.K. (2007). Melanoma in the young: Differences and similarities with adult melanoma. Cancer.

[22] Bauer J., Büttner P., Murali R., Okamoto I., Kolaitis N.A., Landi M.T., Scolyer R.A., Bastian B.C. (2011). BRAF mutations in cutaneous melanoma are independently associated with age, anatomic site of the primary tumor, and the degree of solar elastosis at the primary tumor site. Pigment Cell Melanoma Res..

[23] Matson V., Fessler J., Bao R., Chongsuwat T., Zha Y., Alegre M.-L., Luke J.J., Gajewski T.F. (2018). The commensal microbiome is associated with anti-PD-1 efficacy in metastatic melanoma patients. Science.

[24] Dubin K., Callahan M.K., Ren B., Khanin R., Viale A., Ling L., No D., Gobourne A., Littmann E., Huttenhower C. (2016). Intestinal microbiome analyses identify melanoma patients at risk for checkpoint-blockade-induced colitis. Nat. Commun..

[25] Nagpal R., Mainali R., Ahmadi S., Wang S., Singh R., Kavanagh K., Kitzman D.W., Kushugulova A., Marotta F., Yadav H. (2018). Gut microbiome and aging: Physiological and mechanistic insights. Nutr. Heal. Aging.

[26] Mariat D., Firmesse O., Levenez F., Guimarăes V., Sokol H., Doré J., Corthier G., Furet J.-P. (2009). The Firmicutes/Bacteroidetes ratio of the human microbiota changes with age. BMC Microbiol..

[27] Fuentes E., Fuentes M., Alarcón M., Palomo I. (2017). Immune System Dysfunction in the Elderly. An. Acad. Bras. Cienc..

[28] Simon A.K., Hollander G.A., McMichael A. (2015). Evolution of the immune system in humans from infancy to old age. Proc. R. Soc. B Biol. Sci..

[29] Sadighi Akha A.A. (2018). Aging and the immune system: An overview. J. Immunol. Methods.

[30] van den Brom R.R.H., van der Geest K.S.M., Brouwer E., Hospers G.A.P., Boots A.M.H. (2018). Enhanced expression of PD-1 and other activation markers by CD4+ T cells of young but not old patients with metastatic melanoma. Cancer Immunol. Immunother..

[31] Krauthammer M., Kong Y., Bacchiocchi A., Evans P., Pornputtapong N., Wu C., McCusker J.P., Ma S., Cheng E., Straub R. (2015). Exome sequencing identifies recurrent mutations in NF1 and RASopathy genes in sun-exposed melanomas. Nat. Genet..

[32] Jochems A., Schouwenburg M.G., Leeneman B., Franken M.G., van den Eertwegh A.J.M., Haanen J.B.A.G., Gelderblom H., Uyl-de Groot C.A., Aarts M.J.B., van den Berkmortel F.W.P.J. (2017). Dutch Melanoma Treatment Registry: Quality assurance in the care of patients with metastatic melanoma in the Netherlands. Eur. J. Cancer.

[33] Sender L., Zabokrtsky K.B. (2015). Adolescent and young adult patients with cancer: a milieu of unique features. Nat. Rev. Clin. Oncol..

[34] Edge S.B., Compton C.C. (2010). The American Joint Committee on Cancer: the 7th Edition of the AJCC Cancer Staging Manual and the Future of TNM. Ann. Surg. Oncol..

[35] Majewski W., Stanienda K., Wicherska K., Ulczok R., Wydmanski J. (2015). Treatment Outcome and Prognostic Factors for Malignant Skin Melanoma Treated with Radical Surgery. Asian Pacific J. Cancer Prev..

[36] Farahi J.M., Fazzari M., Braunberger T., Caravaglio J.V., Kretowicz A., Wells K., Dellavalle R.P., Norris D., Alkousakis T. (2018). Gender differences in melanoma prognostic factors. Dermatol. Online J..

[37] Hauschild A., Larkin J., Ribas A., Dréno B., Flaherty K.T., Ascierto P.A., Lewis K.D., McKenna E., Zhu Q., Mun Y. (2018). Modeled Prognostic Subgroups for Survival and Treatment Outcomes in BRAF V600–Mutated Metastatic Melanoma. JAMA Oncol..

[38] Lattanzi M., Lee Y., Simpson D., Moran U., Darvishian F., Kim R.H., Hernando E., Polsky D., Hanniford D., Shapiro R. (2019). Primary Melanoma Histologic Subtype: Impact on Survival and Response to Therapy. JNCI J. Natl. Cancer Inst..

[39] Schemper M., Smith T.L. (1996). A note on quantifying follow-up in studies of failure time. Control. Clin. Trials.

[40] de Glas N.A., Kiderlen M., Vandenbroucke J.P., de Craen A.J.M., Portielje J.E.A., van de Velde C.J.H., Liefers G.-J., Bastiaannet E., Le Cessie S. (2016). Performing Survival Analyses in the Presence of Competing Risks: A Clinical Example in Older Breast Cancer Patients. J. Natl. Cancer Inst..

[41] Putter H., Fiocco M., Geskus R.B. (2007). Tutorial in biostatistics: Competing risks and multi-state models. Stat. Med..

