# Supplementary material for: Age Does Matter in Adolescents and Young Adults versus Older Adults with Advanced Melanoma; A National Cohort Study Comparing Tumor Characteristics, Treatment Pattern, Toxicity and Response
Source: Cancers (Basel). 2020 Jul 27;12(8):2072. doi: 10.3390/cancers12082072 (PMC7464956; doi:10.3390/cancers12082072)
Supplement: Supplementary file 1 [file cancers-12-02072-s001.zip › cancaers-863892-supplementary/cancers-863892-Figure S1_final -1.docx]

**Figure 1.** Types of initial systemic treatment initiated since the diagnosis of advanced melanoma. Cumulative number of targeted therapy and immune checkpoint inhibition initiated over time since July 2013 for adolescents and young adults (AYA, solid line) and older adults (Adult, dotted line) is shown.
