# Supplementary material for: Age Does Matter in Adolescents and Young Adults versus Older Adults with Advanced Melanoma; A National Cohort Study Comparing Tumor Characteristics, Treatment Pattern, Toxicity and Response
Source: Cancers (Basel). 2020 Jul 27;12(8):2072. doi: 10.3390/cancers12082072 (PMC7464956; doi:10.3390/cancers12082072)
Supplement: Supplementary file 1 [file cancers-12-02072-s001.zip › cancaers-863892-supplementary/cancers-863892-supplementary-table.docx]

**Table S1.** Grade 3–4 adverse events following initial systemic treatment. Subtypes of adverse events per treatment type are shown for Adolescents and Young Adults (AYA) and older adults (Adult).

| **Anti-PD-1** | **AYA (*n* = 31)** | **Adult (*n* = 841)** | ***p*-Value** |
| --- | --- | --- | --- |
| Grade III-IV toxicity | 2 (6.5) | 115 (13.7) | 0.25 |
| Colitis | 1 (3.2) | 22 (2.6) |  |
| Intestinal perforation | 0 | 7 (0.8) |  |
| Hepatitis | 0 | 15 (1.8) |  |
| Decline in renal function | 0 | 8 (1.0) |  |
| Nephritis | 0 | 7 (0.8) |  |
| Dyspnea | 0 | 5 (0.6) |  |
| Pneumonia | 1 (3.2) | 9 (1.1) |  |
| Adrenal insufficiency | 0 | 4 (0.5) |  |
| Myelotoxicity | 0 | 2 (0.2) |  |
| Neuropathy | 0 | 2 (0.2) |  |
| Pituitary insufficiency | 0 | 5 (0.6) |  |
| Thyroid insufficiency | 0 | 5 (0.6) |  |
| Fatigue | 0 | 6 (0.7) |  |
| Rash | 0 | 6 (0.7) |  |
| Pruritis | 0 | 2 (0.2) |  |
| Vitiligo | 0 | 6 (0.7) |  |
| Other | 1 (3.2) | 45 (5.4) |  |
| Anti-CTLA-4 | **AYA (*n* = 19)** | **Adult (*n* = 408)** | ***p*-Value** |
| Grade III-IV toxicity | 3 (15.8) | 133 (32.6) | 0.12 |
| Colitis | 0 | 71 (17.4) | 0.046 |
| Intestinal perforation | 0 | 2 (0.5) |  |
| Hepatitis | 0 | 10 (2.5) |  |
| Adrenal insufficiency | 0 | 15 (3.7) |  |
| Myelotoxicity | 0 | 4 (1.0) |  |
| Neuropathy | 0 | 0 |  |
| Pituitary insufficiency | 0 | 27 (6.6) |  |
| Thyroid insufficiency | 1 (5.3) | 13 (3.2) |  |
| Skin toxicity | 1 (5.3) | 11 (2.7) |  |
| Uveitis | 0 | 2 (0.5) |  |
| Other | 1 (5.3) | 22 (5.4) |  |
| Anti-PD-1 & anti-CTLA-4 | **AYA (*n* = 21)** | **Adult (*n* = 169)** | ***p*-Value** |
| Grade III-IV toxicity | 14 (66.7) | 94 (55.6) | 0.34 |
| Colitis | 3 (14.3) | 31 (18.3) |  |
| Intestinal perforation | 0 | 15 (8.9) |  |
| Hepatitis | 9 (42.9) | 36 (21.3) | 0.03 |
| Nephritis | 0 | 4 (2.4) |  |
| Pneumonia | 1 (4.8) | 7 (4.1) |  |
| Adrenal insufficiency | 0 | 2 (1.2) |  |
| Myelotoxicity | 0 | 1 (0.6) |  |
| Neuropathy | 0 | 4 (2.4) |  |
| Pituitary insufficiency | 1 (4.8) | 11 (6.5) |  |
| Thyroid insufficiency | 1 (4.8) | 8 (4.7) |  |
| Fatigue | 0 | 3 (1.8) |  |
| Rash | 1 (4.8) | 9 (5.3) |  |
| Pruritis | 0 | 3 (1.8) |  |
| Vitiligo | 0 | 0 |  |
| Other | 2 (9.5) | 20 (11.8) |  |
| BRAF/MEK inhibitor | **AYA (*n* = 74)** | **Adult (*n* = 1003)** | ***p*-Value** |
| Grade III-IV toxicity | 10 (13.5) | 229 (22.8) | 0.06 |
| Skin malignancy | 0 | 18 (1.8) |  |
| Skin toxicity | 5 (6.8) | 78 (7.8) |  |
| Photosensitivity | 0 | 12 (1.2) |  |
| Palmar-Plantar Erythrodysesthesia | 0 | 16 (1.6) |  |
| Pyrexia | 2 (2.7) | 54 (5.4) |  |
| Hepatitis | 2 (2.7) | 22 (2.2) |  |
| Artralgia | 1 (1.4) | 9 (0.9) |  |
| Alopecia | 0 | 0 |  |
| Decrease left ventriclar function | 0 | 7 (0.7) |  |
| Retinopathy/occlusive retinal vene | 0 | 2 (0.2) |  |
| Other | 4 (5.4) | 83 (8.3) |  |

| 1. 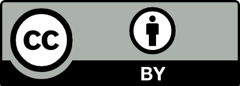 | 1. © 2020 by the authors. Submitted for possible open access publication under the terms and conditions of the Creative Commons Attribution (CC BY) license (http://creativecommons.org/licenses/by/4.0/). |
| --- | --- |
